# The Antimicrobial Resistance–Water–Corporate Interface: Exploring the Connections Between Antimicrobials, Water, and Pollution

**DOI:** 10.3390/tropicalmed10040105

**Published:** 2025-04-12

**Authors:** Jason P. Burnham

**Affiliations:** Division of Infectious Diseases, Washington University School of Medicine, 4523 Clayton Avenue, Campus Box 8051, St. Louis, MO 63110, USA; burnham@wustl.edu; Tel.: +314-454-8821; Fax: +314-454-8687

**Keywords:** antibiotic resistance, water pollution, heavy metal pollution, agricultural pollution, plastic pollution, oil pollution, industrial pollution

## Abstract

Antibiotic resistance is a public health emergency, with ten million deaths estimated annually by the year 2050. Water systems are an important medium for the development and dissemination of antibiotic resistance from a variety of sources, explored in this perspective review. Hospital wastewater and wastewater systems more broadly are breeding grounds for antibiotic resistance because of the nature of their waste and how it is processed. Corporations from various sectors contribute to antibiotic resistance in many direct and indirect ways. Pharmaceutical factory runoff, agricultural antibiotic use, agricultural use of nitrogen fertilizers, heavy metal pollution, air pollution (atmospheric deposition, burning of oil and/or fossil fuels), plastic/microplastic pollution, and oil/petroleum spills/pollution have all been demonstrated to contribute to antibiotic resistance. Mitigation strategies to reduce these pathways to antibiotic resistance are discussed and future directions hypothesized.

## 1. Introduction

Antimicrobial resistance is a public health emergency, with nearly five million antimicrobial-resistant pathogen-related deaths occurring in 2019 and projections of ten million annual deaths occurring by the year 2050 [1,2]. As antimicrobial resistance continues to increase, it puts the very fabric of healthcare systems at risk. Without effective antimicrobials, surgeries, cancer treatment (and administration of immunosuppressing drugs for other non-malignant conditions), organ transplantation, and community-acquired infections could become fatal [3]. Globally, antibiotic-resistant infections are becoming increasingly difficult to treat, are associated with significant healthcare costs, and have high rates of morbidity, mortality, and hospital readmissions [4,5,6,7,8,9]. Specifically, antibiotic-resistant infections have been associated with a 2–3-fold increase in hospital costs, a greater-than-12-day increase in hospital stay [8], 30-day readmission rates exceeding 30% [10], and a 1-year mortality of 35–60% [11].

Antibiotic resistance occurs because of multiple overlapping processes, many of which are related to human activities [12]. Antibiotic use in humans creates selective pressure that increases the prevalence of antibiotic-resistant pathogens [12]. In 2021, outpatient clinic visits accounted for >200 million antibiotic prescriptions, representing >1.5 billion days of antibiotic therapy [13]. An estimated 30–70% of outpatient antibiotic prescriptions may be inappropriate [14,15,16], highlighting a tremendous opportunity to improve antibiotic use, a key part of combating antibiotic resistance. Antibiotic stewardship is one method whereby the use of antibiotics in humans can be limited. Antibiotic stewardship provides direct benefits to individual patients; that is, an individual does not receive an unnecessary antibiotic, thereby eliminating their risk of adverse events from antibiotic use. However, antibiotic stewardship also provides benefit to society more broadly, as effective use of the principles of antibiotic stewardship can potentially reduce the selective pressure of antibiotics that end up in the environment as a downstream consequence of antibiotic use in humans (i.e., antibiotics ending up in wastewater systems).

However, antibiotics are not only used in humans. Antibiotics, fungicides, and pesticides are all utilized in agriculture and contribute to the development of antibiotic resistance. These antibiotics, fungicides, and pesticides, whether entering the environment directly via humans or indirectly from animals and agricultural processes, disseminate themselves globally, including into water systems which are central to human and animal use and consumption.

**Figure 1:**
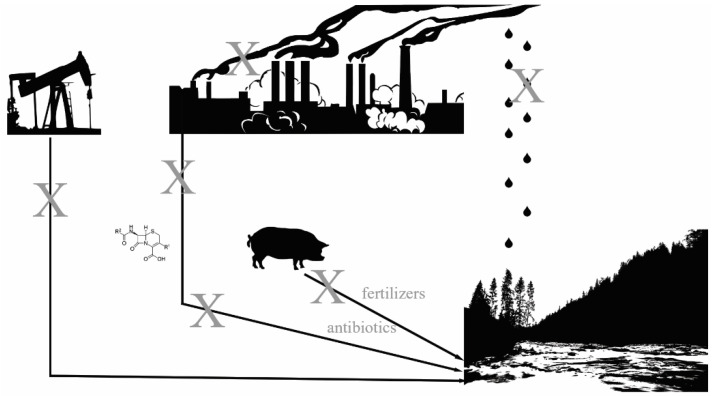
Sources of antibiotic resistance moieties. Xs represent areas to break transmission of antibiotic resistance in the environment, including reducing oil production and spills, reducing pharmaceutical use and waste, reducing fertilizer and antibiotic use in agriculture, and reducing air and water pollution into water systems.

Water systems are increasingly recognized as breeding grounds for antimicrobial resistance, particularly those systems into which human pollution enters. Pollution of all kinds can contribute to antibiotic resistance, including sewage, plastics, heavy metals, pharmaceuticals, oil/petroleum, and nitrogen fertilizers (see Figure 1) [17,18,19,20,21,22,23,24,25,26,27]. This paper will discuss the interface between water, corporations, and antimicrobial resistance.

### 1.1. Water and Antimicrobial Resistance

Water sources are increasingly recognized as being conducive to the development of antimicrobial resistance, including sinks/hospitals, hospital wastewater, and wastewater systems more generally [19,28,29,30,31,32,33,34,35,36]. In a study of hospital intensive care unit sinks and water in the United States and Pakistan, Diorio-Toth et al. [28] found a high burden of antibiotic-resistant bacteria, including *Pseudomonas*, *Acinetobacter*, and *Stenotrophomonas*. These antibiotic-resistant bacteria persisted in the hospital environment for up to two years [28]. Kwon et al. [30] demonstrated that antibiotic-resistant bacteria, including *Pseudomonas*, can establish themselves even in newly built hospital buildings, with the highest burden of organisms related to sink drains. In addition, Kwon et al. [30] found through genomic sequencing that the organisms establishing themselves in sink drains in the new hospital building were directly related to patient infections, as the organisms (confirmed by genomic analyses) were identified in blood cultures from infected patients. In addition to being a biohazard risk within a patient’s room, these antibiotic-resistant organisms have been shown to be highly mobile. In a study by Mathers et al. [31] using green fluorescent protein to track the mobility of bacteria in hospital water systems, it was found that *Escherichia coli* biofilms were able to extend through sink drains, which subsequently led to their spread by droplet dispersion during operation of sinks. In addition to bacteria spreading from sinks and throughout the rooms, Mathers et al. [33] also demonstrated that antibiotic-resistant bacteria can spread from the patient directly to the in-room water system. Mathers et al. [31] also demonstrated that the antibiotic-resistant bacteria could travel retrograde via common pipe systems. In a further study outside the hospital, Mathers et al. [32] tracked *Klebsiella pneumoniae* and its carbapenemase (i.e., KPC) from the hospital to wastewater discharges and a municipal wastewater treatment plant. They found that *K. pneumoniae* and KPC were detectable in hospital wastewater and the principal stages of wastewater treatment, but were eliminated by UV disinfection before reaching the final effluent [32].

In addition to the dissemination of antibiotic-resistant bacteria within and adjacent to hospital systems, pharmaceutical manufacturing runoff contributes molecular compounds that lead to antimicrobial resistance [37,38]. The putative solutions to reducing these problems are myriad. Generally speaking, antibiotic stewardship in healthcare could lead to reductions in antibiotic resistance by putting fewer antibiotics into the environment to which bacteria could become resistant [12]. Given that high antibiotic exposure in hospital wards trickles down to antibiotic resistance in wastewater systems [39], it is possible that the fewer antibiotics that are prescribed by health providers, the fewer opportunities there are for those antibiotics to leach or be directly shed (by humans or hospitals) into water systems.

Another aspect of antibiotic removal puts the onus on hospitals to degrade the antibiotics prior to their entry into water systems. Hospital water cleaning systems have a differential efficiency for antibiotic removal, with some antibiotics harder to remove, such as fluoroquinolones [40]. Some strategies with promise for future use in removal of antibiotics from hospital wastewater include photolytic degradation [41] and electro-Fenton processes with various novel catalysts [42,43].

In addition to antibiotics themselves, hospital wastewater systems also contain high concentrations of antibiotic resistance genes, which are important in antibiotic resistance spread, as they can be readily shared between bacteria of different genus and species via plasmids and transposons. Various strategies have been used to attempt to reduce the concentration of antibiotic resistance genes in water systems, including ozonation, UV and chlorine, and various bioelectrochemical methods [44,45,46,47,48]. To date, no antibiotic degradation or antibiotic resistance gene elimination method has emerged and been widely adopted, but research efforts should focus on choosing cost-effective, environmentally friendly strategies to protect human and environmental health.

All of the sources of compounds that contribute to antibiotic resistance mentioned above relate back to a corporate responsibility to preventing antimicrobial resistance, whether that is hospital-based corporations, health insurance companies, pharmaceutical manufacturers, or agriculture corporations—there is a One Health responsibility for each of these entities to reduce their antimicrobial resistance footprint not just for humans, but the environment and all living things in it.

### 1.2. Corporations and Antimicrobial Resistance

As mentioned above, corporations have a responsibility to protect the environment from the introduction of antimicrobial resistance, as this downstream side effect of their waste will cause morbidity and mortality for humans and animals alike. Corporation-related antimicrobial resistance has been documented in numerous instances (Table 1). Pharmaceutical factory runoff in India has been shown to be related to antimicrobial resistance, with high levels of antibiotics in rivers in close proximity to factories which produce pharmaceutical waste products [49]. Indeed, polluted water sources have been speculated to have the potential to lead to pandemic proportions of antimicrobial resistance [50]. A recent study of the world’s rivers found that pharmaceutical pollution is a worldwide problem, with no continent spared [51]. The areas with the highest levels of pharmaceutical contamination include sub-Saharan Africa, south Asia, and South America [51]. Global problems such as these will require global solutions.

### 1.3. Agriculture and Antibiotics

Antibiotics have long been recognized as growth promoters to increase meat yield in animals [72]. The Food and Drug Administration banned the use of antibiotics important for human medicine solely as growth promoting agents in animal agriculture in 2017, but studies have shown that this practice persists in the US [73]. Medically important antibiotics are also used globally, with an increase in recent years, despite well-accepted data that link animal agriculture antibiotic use and the development of antibiotic resistance in humans [52]. The use of antibiotics in animal agriculture ties into the water system, as these antibiotics end up in water supplies, where they can promote the development of antibiotic resistance [53,54]. As the practice of antibiotic use for growth promotion increases meat yield and corporate profit at the overall expense of human health, additional measures must be taken to end such antibiotic misuse.

In addition to the antibiotics that are used to increase meat yield, farming and agriculture also participate in dumping of nitrogenous wastes, which increase antibiotic resistance by multiple pathways [17,18]. More generally, nitrates are dumped into water systems in huge amounts each year, with nearly 200 million pounds of toxic pollution dumped into US waterways in 2020 alone [74]. Practices such as this must be curbed to slow the tide of not only antibiotic resistance but also of the destruction of our critical freshwater ecosystems.

### 1.4. Pollution and Antibiotic Resistance

Pollution comes in many flavors in addition to those mentioned above (direct antibiotic and pharmaceutical pollution, indirect antibiotic and pharmaceutical pollution/contamination of groundwater), including heavy metal pollution. Historically, companies have been responsible for massive heavy metal polluting events, and their effects are still being felt. Three of the largest heavy metal poisoning events include those perpetrated by the Chisso Corporation in Japan (mercury pollution of water in Minamata Bay), Sandoz in Switzerland (mercury and pesticides dumped into Rhine River), and Pacific Gas and Electric in Hinkley, California (release of hexavalent chromium into unlined ponds resulting in groundwater contamination). Given the association between heavy metals and development of antibiotic resistance (as well as many other human, animal, and plant direct and indirect toxicities), companies have a humanitarian and environmental responsibility to prevent pollution and spills now and in the future. In an Australian study of subtidal surface sediments and sediment cores, Coates-Marnane et al. [55] demonstrated that flooding events resulted in increases in concentrations of zinc, copper, and lead in coastal waters. In a study of Scottish soil samples, Knapp et al. [21] found that the abundance of antibiotic resistance genes in soil was positively correlated with soil copper, chromium, nickel, lead, and iron levels. In an analysis of published studies, Seiler and Berendonk [22] concluded that currently available data suggests a link between heavy metal pollution and antibiotic resistance, including the possibility for co-selection, i.e., for antibiotic resistance and heavy metal resistance to have a multiplicative effect greater than the individual resistances to antibiotics/heavy metals alone.

### 1.5. Air Pollution and Antibiotic Resistance

Recent studies have shown that air pollution contributes to antimicrobial resistance [56,57,58,59]. Though not directly polluting water systems, as happens with direct pollution like the 200 million pounds of nitrate waste in the US that was mentioned above, the aerosols that contribute to and harbor antibiotic resistance can eventually end up in water systems through atmospheric deposition. Atmospheric deposition is a term describing the transport of gases and particles from the atmosphere to terrestrial and aquatic surfaces.

The top global polluters are largely oil and/or fossil fuel based companies, with the top nine polluters in 2023 including Peabody Energy, Kuwait Petroleum Corp, ConocoPhillips, Chevron, Saudi Aramco, ExxonMobil, BP, National Iranian Oil Co, and Royal Dutch Shell [60]. The acquisition, processing, and use of the oil and fossil fuels produced by these companies significantly contributes to air pollution globally, as well as the production of greenhouse gases, which directly warm and alter the climate. Climate change is the worst public health emergency of the 21st century [75], and climate change in and of itself is associated with antimicrobial resistance [76]. These (and all polluting companies) must be held accountable for their emissions which harm the planet and human health.

### 1.6. Plastics, Water, and Antibiotic Resistance

The global burden of plastic and microplastic pollution is increasing. Relevant to antibiotic resistance and water, in aquatic environments, microplastics can be substrates for antimicrobial resistance development [23,61,62,63,64,65,66,67,68,69,70]. Plastic and microplastic pollution originates with corporations, and some of the worst global plastic polluters include Coca-Cola, Pepsico, Nestlé, Unilever, and Mondeléz International [60]. Plastics and microplastics are found in virtually every corner of every biome on Earth and their continued spread and accumulation must be curbed to help prevent antibiotic resistance and promote planetary health.

### 1.7. Oil/Petroleum and Antibiotic Resistance

Some of the worst environmental disasters in history have been related to the oil/petroleum industry, including Gulf War-related fires and oil dumping into the Persian Gulf, the Pemex Ixtoc 1 oil spill in the Gulf of Mexico, and the 2010 BP oil spill (also known as Deepwater Horizon). In an analysis of soil bacteria from the Arabian Sea and Bay of Bengal coastlines, investigators found antibiotic-resistant organisms relevant to human health that were related to oil spills and biohazardous pollution entering the waters [27]. Following the 2010 BP oil spill, investigators found that antibiotic resistance in bacterial isolates from bottlenose dolphins was higher than expected, attributed to the oil spill [26]. In addition, genes for multidrug resistance efflux genes were expressed at a percentage as high as that in wastewater (a known reservoir for antibiotic resistance) in samples taken from marine sediments exposed to the 2010 BP oil spill [71]. The effects of these oil spills linger into the present and corporations have a responsibility not only to maximize prevention of future spills, but also to remediate those whose damage persists.

### 1.8. Mitigation: Where Do We Go from Here? What Can We Do?

Global problems require global solutions, and some progress is being made in identifying processes that may help minimize the impacts of various of the forms of antibiotic-resistance promoting pollution mentioned above.

### 1.9. Antibiotic Resistance Genes and Antibiotic-Resistant Organisms

Biological wastewater treatment plays a large role in protecting humans against transmission of waterborne disease, including, but not limited to, antibiotic-resistant infections. Wastewater is a known reservoir for antibiotic resistance. The typical processes used to treat wastewater are energy inefficient, but biologically effective. A recent study showed that anaerobic-aerobic sequence reactors (as compared to standard active aeration processes such as activated sludge) effectively reduced levels of antibiotic resistance genes in domestic wastewater with a concomitant 32% reduction in energy use (as compared to industry standard) [77]. Additional studies have also demonstrated the validity and favorability of using anaerobic processes to reduce antibiotic resistance genes in wastewater [78].

Another potentially more environmentally friendly way of moving antibiotic resistance genes from wastewater is referred to as a constructed wetland, which refers to an artificial wetland which simulates the natural processes to treat domestic and livestock wastewaters. An integrated surface flow constructed wetland is utilized in wastewater treatment as a replacement for a centralized wastewater treatment plant. Constructed wetlands can be effective in rural and less developed areas because of reduced cost and available undeveloped lands upon which to construct the wetland. Fang et al. [79] investigated the effectiveness of antibiotic resistance gene removal for an integrated surface flow constructed wetland in rural China that began operating in 2005. They found that this constructed wetland had a 60–80% efficiency in removing antibiotic resistance genes, an efficiency that varied based on time of year [79]. However, certain antibiotic resistance genes were found to increase in this integrated surface flow constructed wetland schema and further information is needed to determine the overall environmental impact relative to traditional centralized wastewater plants. In addition, the type of constructed wetland also modifies the efficiency of antibiotic resistance gene removal. In a 2016 study, Chen et al. [80] found that flow configuration and plant species affected antibiotic resistance gene removal.

Nanomaterials have a more nuanced role in mitigation of antibiotic resistance genes, as some studies show benefit and others show potentially harmful downstream effects. Therefore, nanomaterials are likely not a good target for antibiotic resistance gene mitigation at this time until further studies can be performed.

Coagulation is an active method to remove colloidal particles in water and is broadly used in wastewater treatment plants to improve water quality and remove contaminants. Coagulation can improve water turbidity, color, and reduce organic matter and heavy metals. Though not specifically designed to remove antibiotic resistance genes from wastewater, studies in recent years have investigated the effects of the already widespread process of coagulation on antibiotic resistance genes in wastewater. In an investigation by Li et al. [81], which examined removal of five antibiotic resistance genes in a wastewater treatment plant in China by coagulation with the coagulants FeCl_3_ and polyferric chloride, it was found that wastewater effluents had 0.5-log to 3.1-log reductions in antibiotic resistance genes. The authors concluded that while these coagulants need further study, it was a promising direction in antibiotic resistance gene removal [81]. In 2022, Yue Jian et al. [82] investigated three common coagulants, polyaluminum chloride, polyaluminum sulfate, and aluminum hydroxide ion, for their effect on the removal of microorganisms and antibiotic resistance genes from swine wastewater. The authors found that all three coagulants were effective in removing both microorganisms and antibiotic resistance genes from swine wastewater, which, coupled with results from other studies, had suggested a potential broad applicability of coagulants as a means to remove antibiotic resistance genes [82]. However, a recent study calls the efficacy of coagulation as a means for antibiotic resistance gene removal into doubt. In 2024, Fazhu Wu et al. [83] set up a model wastewater treatment plant which utilized two common coagulants—polyaluminum chloride and ferric chloride—to treat wastewater from a municipal wastewater treatment plant in Hefei, China. During the first phase of the study, removal efficiencies of both coagulants were over 90% [83]. However, after four days of storage of the samples (a time interval that could feasibly occur in non-laboratory settings, i.e., in wastewater treatment plants), the level of antibiotic-resistant bacteria in the samples increased from 6 to 138 fold, as compared to the initial levels found in the secondary effluent from the wastewater treatment plant [83]. The authors posited that this increase was potentially caused by several factors, including enhanced cell–cell contact by bacteria during coagulant-induced bacterial aggregation, prolonged incubation times in the wastewater treatment plant model, typical operating temperatures in wastewater treatment (i.e., ones that could promote bacterial growth and increase enzymatic activity such as that necessary for conjugative transfer of antibiotic resistance plasmids), and a coagulant-induced gene expression that results in increased conjugation efficiency [83]. Though coagulation has some potential as a future method of removal of antibiotic-resistant bacteria and antibiotic resistance genes from wastewater, the full process of wastewater treatment is complex and further studies are needed to understand how modifications to any stage of wastewater treatment change the levels of antibiotic-resistant bacteria and antibiotic resistance genes in the final wastewater effluent.

On the patient-facing side of antibiotic resistance prevention, Mathers et al. [34] tested an intervention to prevent antibiotic-resistant organism infections in hospitalized patients. In this study, an intervention was carried out in intensive care units wherein covers were placed on in-room waste disposal systems and sink trap heating and vibration devices were installed [34]. With this intervention, the odds ratio for acquisition of the antibiotic resistance organism from the hospital water system was 0.51, which represented a significant decrease [34]. Thus, even in situations where antibiotic-resistant organisms are already established, there are at least methods for potentially reducing their morbidity and mortality on individual patients.

### 1.10. Plastics

As discussed above, plastics pollution is an increasing problem globally and provides a substrate upon which antibiotic resistance can develop and transfer between organisms [23,61,62].

Removal of plastics and microplastics from the environment can be accomplished by many avenues. By removing these from the environment and water systems, there will be fewer opportunities/substrates for the development and spread of antibiotic resistance. Some potential macro-level methods to reduce environmental plastics include policy development, which encourages reductions in the use of plastic-containing products, legislation to reduce plastics, such as prohibiting single-use plastic bags, and tax systems for plastic-containing products [62]. On a micro level, studies have shown that certain membrane filters, i.e., a dynamic membrane, can result in significant reductions in low-density, non-degradable micro-particles such as plastics from wastewater effluent [84]. Another putative method for plastic removal is the utilization of hybrid silica gels, but this method requires practical studies to determine its efficacy when deployed outside a laboratory setting [85]. Another study showed that alum, a coagulant, resulted in significant reductions in plastics in simulated wastewater [86]. As mentioned above, coagulation has been shown to remove antibiotic resistance genes from wastewater. With the study of alum showing reductions in plastics in water systems [86], it is possible that some coagulants may have a dual role to remove both antibiotic resistance genes and plastics from wastewater systems, but further study is required.

## 2. Conclusions

Myriad types of pollution, at the hands of corporations, degrade the environment and contribute to antibiotic resistance, which has profound implications for human health. This paper has taken an anthropocentric viewpoint, focusing primarily on the human health implications of water and water-related pollutants and antibiotic resistance. However, the effects of these pollutants and antibiotic resistance on other organisms are likely myriad, and, similarly to human-related effects (or perhaps to an even greater degree), likely understudied and underreported. Pollution, climate change, and antibiotic resistance are critical issues of the twenty-first century and their unmitigated progression will result in harm to human life, and likely all other life on Earth. We must take action at every level to reverse the tide of these interwoven catastrophes on the horizon, starting with holding corporations accountable for their direct and indirect damage. To begin, corporations must transparently report their pollution and environmental impacts. This will be a good place to start, but a better metric to measure environmental antibiotic resistance impacts from corporation-related pollution must be developed and standardized in order to hold corporations accountable. We have a duty to both to protect the health of our fellow humans and our planet.

## 3. Future Directions

As with all things in healthcare and climate change, the interrelationships between antibiotic resistance, water systems, corporations, and the environment are extremely complex (Figure 1). It is possible, though improbable, that there is one single approach that will be globally successful, as local, regional, and national contexts are undoubtedly unique and will pose unique challenges. In order to solve these challenges, a strategy with which to move forward will have to be adaptable, but will have some broad, overarching elements which are non-negotiable, and those are human and environmental health.

A broad approach for minimizing antibiotic resistance in the realm of water systems must include the elements discussed below. The most important of all steps to be taken is addressing corporate responsibility. How this is accomplished will almost certainly vary based on local contextual factors. Whether this is via monetary incentives, new laws and/or regulations, or grassroots campaigns from individuals to influence corporate practice, corporations must calculate their antibiotic resistance footprint and take steps to minimize it in order to protect human and environmental health. In instances where corporate introductions of antibiotic resistance into the environment (whether direct via antibiotics, pharmaceuticals, or indirect via pollution) are unavoidable, corporations must identify ways to mitigate these introductions. Some strategies for reducing these introductions of antibiotic resistance (direct or indirect) have been reviewed above, but may include direct removal or recapture of antibiotics/pollutants.

At an international level, a decreased emphasis on corporate profit and an increased focus on sustainability and protection of humanity and the environment will reduce incentives to produce products/services at the expense of planetary and human health. Other high-level changes must include reducing ties between corporations and governments to reduce the insidious influence of corporations on laws/regulations that permit pollution and antibiotic resistance in the environment. Historically, there has been a push for individual-level responsibility for consumption and the use of products that contribute to pollution, but, as outlined in this paper, the largest polluters and those driving consumption that produce environmental pollutants, greenhouse gases, and antibiotic resistance are corporations. Individual decisions may have some impact on these metrics, but corporations must be held accountable to have a measurable impact and rescue humanity and the environment from the march of environmental destruction under which we are presently suffering. There is hope, and we all must hold our governments responsible for their decisions and ties to corporations which harm us all.

## Figures and Tables

**Table 1:** Corporate niduses of direct and indirect antibiotic resistance.

Nidus of Antibiotic Resistance	Direct or Indirect Antibiotic Resistance?	Corporate Contributors	References
Antibiotic use (humans)	Direct	Healthcare	[12]
Antibiotic use (agricultural)	Direct	Agriculture	[17,18,52,53,54]
Pharmaceutical waste and residues	Direct and indirect	Pharma	[37,38,49,51]
Heavy metals	Indirect	Chemical and oil companies	[21,22,55]
Air pollution	Indirect	Oil and gas companies	[56,57,58,59,60]
Plastics	Indirect	Oil, gas, plastic companies	[23,60,61,62,63,64,65,66,67,68,69,70]
Oil spills	Indirect	Oil companies	[26,27,71]
